# Enhancing Cervical Pre-Cancerous Classification Using Advanced Vision Transformer

**DOI:** 10.3390/diagnostics13182884

**Published:** 2023-09-08

**Authors:** Manal Darwish, Mohamad Ziad Altabel, Rahib H. Abiyev

**Affiliations:** Department of Computer Engineering, Applied Artificial Intelligence Research Centre, Near East University, Mersin 10, 99138 Nicosia, Turkey; manal.khaled.darwish@gmail.com (M.D.); mohamadziad.altobel@neu.edu.tr (M.Z.A.)

**Keywords:** cervical cancer, vision transformer, ViT, shifted patch tokenization

## Abstract

One of the most common types of cancer among in women is cervical cancer. Incidence and fatality rates are steadily rising, particularly in developing nations, due to a lack of screening facilities, experienced specialists, and public awareness. Visual inspection is used to screen for cervical cancer after the application of acetic acid (VIA), histopathology test, Papanicolaou (Pap) test, and human papillomavirus (HPV) test. The goal of this research is to employ a vision transformer (ViT) enhanced with shifted patch tokenization (SPT) techniques to create an integrated and robust system for automatic cervix-type identification. A vision transformer enhanced with shifted patch tokenization is used in this work to learn the distinct features between the three different cervical pre-cancerous types. The model was trained and tested on 8215 colposcopy images of the three types, obtained from the publicly available mobile-ODT dataset. The model was tested on 30% of the whole dataset and it showed a good generalization capability of 91% accuracy. The state-of-the art comparison indicated the outperformance of our model. The experimental results show that the suggested system can be employed as a decision support tool in the detection of the cervical pre-cancer transformation zone, particularly in low-resource settings with limited experience and resources.

## 1. Introduction

Cervical cancer is the second most prevalent cancer affecting the female reproductive system [1]. To date, it continues to cause significant morbidity and mortality in developing countries such as China. This has a profound impact on the overall health and quality of life of women [2]. Cervical cancer originates in the cells that line the cervix, which is the lower portion of the uterus or womb. The cervix connects the upper part of the uterus where a fetus develops to the vagina or birth canal. Cancer develops when cells in the body begin to grow uncontrollably [1]. To gain further insight into how cancer originates, and spreads, further research and studies may be necessary.

The cervix is comprises two distinct parts, each covered by different types of cells. The endocervix, which is the opening of the cervix that leads to the uterus and is covered by glandular cells. The exocervix (also known as the ectocervix) is the outer part of the cervix, which is visible during a speculum exam and is covered by squamous cells [1,3].

The location in the cervix where the glandular and squamous cells meet is referred to as the transformation zone. The exact position of the transformation zone can shift as a woman ages or after giving birth. The majority of cervical cancers develop from cells within the transformation zone.

**Figure 1:**
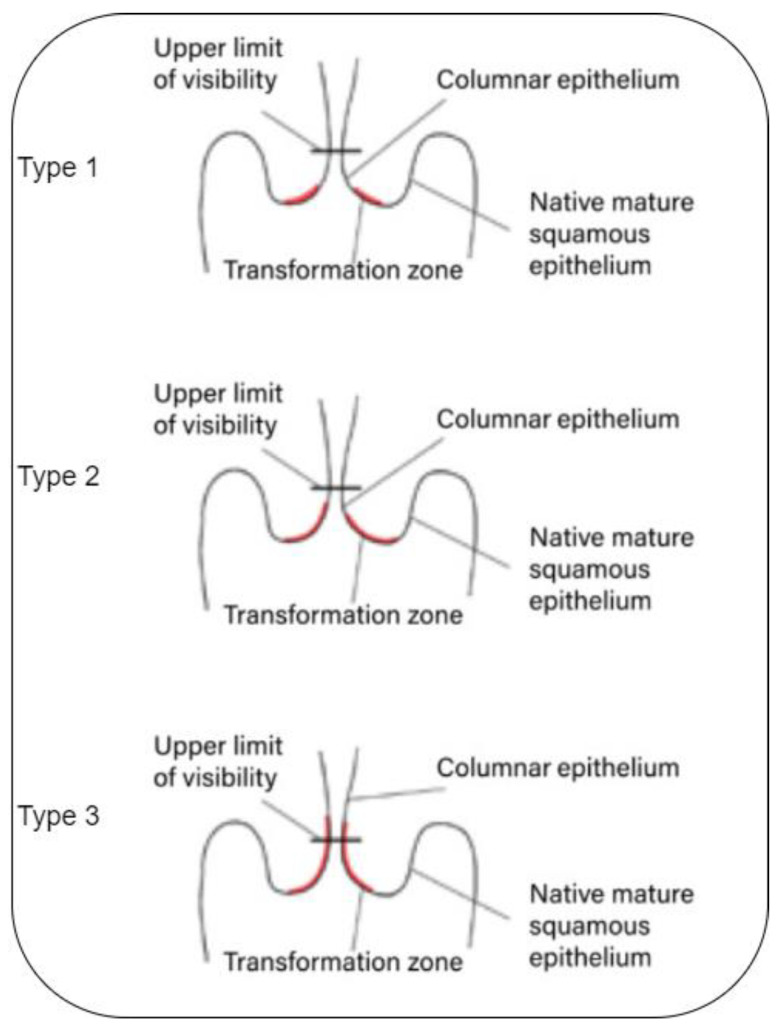
Transformation zone [17].

The transformation zone cells do not abruptly transform into cancerous cells. Instead, the cervical cells typically experience a gradual progression from normal to abnormal changes, which are referred to as pre-cancerous [1,2,3,4,5]. These pre-cancer changes can be graded via colposcopies divided into three different types or grades (See Figure 1):

Type 1: Cervical intraepithelial neoplasia (CIN);

Type 2: Squamous intraepithelial lesion (SIL);

Type 3: Dysplasia.

Cervical cancer is known to originate from pre-cancerous cells, but not all women with pre-cancerous cervical cells will develop the disease [2,3]. In fact, a majority of women with pre-cancerous cells will not require any treatment, as the cells often regress spontaneously. However, for some women, pre-cancerous cells may progress to invasive cancer. The timely treatment of cervical pre-cancers has demonstrated a significant impact in the prevention of cervical cancer, with nearly all cases being preventable through early detection and appropriate medical intervention.

Numerous factors can influence the precision of cervical biopsies in clinical practice, such as the colposcopist’s experience, lesion location, size, depth, and the menstrual status of the patient [1,6]. Even in the hands of experienced colposcopists, the sensitivity of colposcopy can vary significantly. Consequently, enhancing the accuracy of colposcopy is a critical concern in screening cervical cancer.

Deep learning and artificial intelligence in general have positively affected computer-assisted medical diagnosis, especially with the availability of large quantities of clinical data that can help artificial intelligence models to achieve remarkable performance on various medical tasks [7]. Research has indicated that medical artificial intelligence (AI) and computer-assisted diagnosis (CAD) may aid in the detection of cervical lesions and enhance diagnostic accuracy through the use of deep learning and medical image processing technology, combined with possible physiological and pathological knowledge [8,9,10]. Investigations in the areas of optical coherence tomography [11], radiology [12], computerized tomography scan [13], colonoscopy [14], and pathologic slides [9] have suggested that computer algorithms, trained on a large number of medical images in a convolutional neural network (CNN), may approach or even exceed the diagnostic accuracy of clinicians.

The objective of conducting cervical cancer screening is to detect any signs of pre-cancer or cancer at an early stage when it can be more effectively treated and cured. By undergoing regular screening, individuals can potentially avoid developing cervical cancer altogether and ultimately, save lives. Hence, screening the transformation zone and accurately identifying the type of the cervix can be a key element in managing how the change will occur and whether an abnormality/malignancy transformation may happen. Thus, in this work, we aim to identify the type of cervix using a Vision Transformer ViT-based model trained on colposcopy images of the three different types, obtained from the Kaggle Public cervical cancer screening dataset [15]. For this purpose, we employ the use of ViT architecture with enhanced features such as Shifted Patch Tokenization and Locality Inductive Bias adopted from [16], which can help such model improve its performance even if trained on small datasets [16]. The objective of the study was to develop a new colposcopy-based diagnostic system that could effectively and precisely detect/identify the type of the pre-cancerous transformation zone in a colposcopy raw image which can be a helpful tool for medical professionals to better prevent the occurrence of cervical cancer. The contributions of the paper include:

The fusion of two powerful technologies- improved vision transformers and shifted patch tokenization is proposed for cervical cancer classification.

- The structure of the cervical image classification system is proposed. The integration of transformers with a shifted patch tokenization mechanism is presented and a finer granularity of analysis is achieved.
- The presented system is designed using a cervical image data set and implemented for diagnosing cervical cancer. The synergy of vision transformers and shifted patch tokenization culminates in an unprecedented methodology for classifying three distinct types of the cervical pre-cancerous colposcopy images.
- The proposed system has shown better accuracy performance in comparison with other models which improved the effectiveness of cervical cancer classification system.

This paper is structured as follows: Section 1 is an introduction of the work describing the objectives and motivations behind this study. Section 2 is the literature review part. Section 3 is the materials and methods where ViT and SPT are discussed. Section 4 is the model development and parameters tuning, while Section 5 discusses the results and findings of the model. Section 6 is the discussion and results comparison, and, finally, Section 7 is the conclusion.

## 2. Related Works

In 2009, Acosta et al. [18] utilized the K-NN algorithm to differentiate normal and abnormal cervical tissue in aceto-white patterns and obtained a sensitivity of 71% and specificity of 59%. Later, Asiedu et al. [19] achieved a sensitivity, specificity, and accuracy of 81.3%, 78.6%, and 80.0%, respectively, in distinguishing CIN+ and benign tissues. Liming Hu et al. [20] established a cohort and conducted a 7-year follow-up using images captured via cervicography to train and validate a deep learning algorithm, which demonstrated higher accuracy compared to pap smear. Additionally, Bing Bai et al. [21] applied the K-means algorithm to automatically segment the cervical region, indicating the feasibility of cervical segmentation.

In a study more related to ours, [22] proposed a deep learning approach to classify colposcopy images into three types (type 1, type 2, and type 3) for automatic detection of cervical cancer. The researchers used a widely used cervical screening dataset to train and test their model. They introduced a deep network named “Colposcopy Ensemble Network” (CYENET) which outperformed other models such as VGG16 and VGG19 in terms of accuracy. The CYENET achieved an accuracy of 92.3% in their testing phase. However, it is important to note that the number of testing images used in this study was relatively low (1884), which may have contributed to the high accuracy.

Furthermore, Mustafa and Dauda [23] proposed a deep learning method for classifying cervical images into healthy or cancerous using three different deep convolutional neural networks (DCNNs) with various optimizers, including stochastic gradient descent (SGD), Root Mean Square Propagation (RMSprop), and Adaptive Moment Estimation (Adam). To discover the ideal optimizer for obtaining optimal network performance, the model was trained and tested using malignant and healthy cervical images.

The research papers [24,25,26,27] used deep learning-based approaches for classification cervical cancers. The study [24] introduces an innovative CAD framework termed “CerCan·Net” [24] for automating cervical cancer diagnosis. CerCan·Net adopts a unique approach by employing three lightweight CNN architectures—MobileNet, DarkNet-19, and ResNet-18—with fewer parameters and shallower depths compared to traditional models. This strategic selection aims to streamline the classification process and reduce complexity. A key to CerCan·Net’s efficacy is the utilization of transfer learning, harnessing the power of deep features extracted from the last three layers of each CNN, rather than relying solely on a single layer’s features. This approach allows for a more comprehensive representation of the data’s intricacies.

In another approach, a study [25] delves into a crucial and sensitive issue: cervical cancer’s profound impact on medical, psychological, and social facets of women’s lives. Focused on the dataset from the Intel and MobileODT Cervical Cancer Screening competition on Kaggle, the study tackles both the multi-class classification problem and the multi-label classification problem while incorporating image size optimization.

The authors start by highlighting the urgency of the cervical cancer problem and its multidimensional implications. They employ a dataset with updated six-class labels to formulate their investigations. Notably, they employ state-of-the-art deep neural networks (DNNs), including standard DNNs like MobileNetV2 and NASNetMobile, as well as the EfficientNetB0 model, to address these challenges.

In the realm of multi-class classification, the authors ingeniously leverage pretraining on the ImageNet dataset to initialize standard DNNs. Their results manifest that this approach yields improved metrics, highlighting the potential of utilizing compact DNN versions. This insight holds significance, potentially facilitating resource-efficient implementations without compromising performance.

Transitioning to the multi-label classification problem, the study adopts the EfficientNetB0 model as a case study. The authors explore the efficacy of enhancing metrics through image size optimization—a noteworthy pursuit in medical imaging. Through meticulous experimentation, they ascertain that tweaking the input image size produces tangible improvements. Notably, they achieve a notable enhancement of mean AUC values, a 2.7–2.8% increase compared to conventional 224 × 224 pixel sizes. Importantly, this improvement is observed within a range of standard deviations (0.3–1.8%), enhancing the reliability of their findings.

A particularly intriguing facet of the study is the proposal of an innovative strategy for image size optimization. By amalgamating metrics derived from diverse DNN training regimes (with and without data augmentation) and validation/testing procedures for varying image sizes, the authors present an effective approach. Extrapolating trends from these metrics’ variations, they lay the foundation for potential image size optimization in diverse contexts beyond cervical cancer classification.

## 3. Materials and Methods

### 3.1. Vision Transformers (ViTs)

Transformers, originally developed for natural language processing (NLP), have been found to be promising for image identification and understanding [28]. However, due to the large number of pixels in images, it was challenging to apply transformers to this task since every pixel relates to every other pixel in a self-attention mechanism, unlike text [29]. Recent studies have proposed various approaches to incorporating transformers into computer vision, including combining convolutional neural networks (CNNs) with self-attention [30], and employing transformers on top of CNNs to process visual tokens and generate powerful features [31].

One notable contribution in this area is the Vision Transformer (ViT) introduced by Dosovitskiy et al. [32], which partitions images into patches that are treated as tokens and converted into embeddings to be processed by a transformer encoder. This approach allows models to learn image structure independently, and class labels for the image can be predicted [32,33]. The ViT encoder consists of several blocks, each with a normalization layer to adjust to training image differences, a multi-head attention layer to create attention maps, and a multi-layer perceptron (MLP) to process the embeddings. The last MLP block, known as the MLP head, produces the output, which can be subjected to Softmax to produce the probabilities of the categorization labels [34].

This structure enables the ViT to retain more spatial information than CNNs, which can help it learn high-quality intermediate representations with large amounts of data. Attention maps, similar to those found in conventional computer vision literature (e.g., saliency maps and alpha-matting) [34,35], are created from the embedded visual tokens, allowing the network to focus on the most critical areas of the image, such as objects. The second layer of the MLP classification network usually consists of two layers of Gaussian Error Linear Units (GELU) [34].

### 3.2. Shifted Patch Tokenization (SPT)

The Vision Transformer (ViT) [32] has been shown to be a data-hungry model, requiring pretraining on large datasets such as JFT300M and fine-tuning on medium-sized datasets like ImageNet to surpass state-of-the-art convolutional neural network (CNN) approaches. However, when fine-tuned on small datasets, ViT’s performance suffers due to the lack of locality inductive bias in its self-attention layer. In contrast, CNNs leverage spatial sliding windows to achieve better results with smaller datasets. Nonetheless, the conventional ViT can be outperformed by CNNs when dealing with small datasets.

To address this issue, a recent study [16] proposes a modified ViT architecture that incorporates shifted patch tokenization (SPT) and locality self-attention (LSA) to enhance its performance on small datasets. The SPT technique involves moving the image diagonally and combining the original and shifted images to extract patches, which are then flattened and projected after normalization. The proposed architecture has been shown to perform better than CNNs and regular ViT on small datasets, as demonstrated by the Cervical screening dataset used in the study. The application of SPT and LSA allows ViT to effectively capture local correlations between image pixels, leading to improved performance even with limited data.

**Figure 2:**
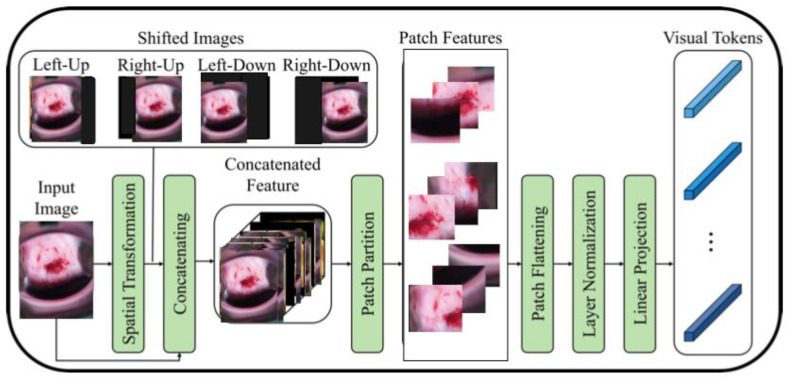
The process of shifted patched tokenization of one Type 2 colposcopy image.

**Figure 3:**
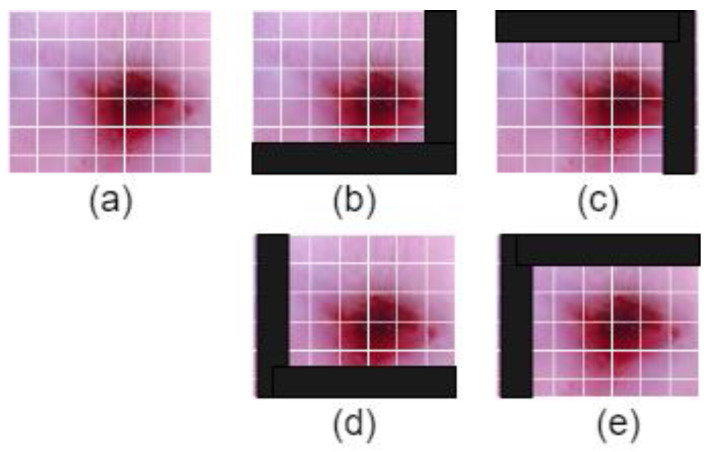
The shifted images of one Living room images during the process of shifted patch tokenization. (**a**) Original image, (**b**) left-up, (**c**) left-down, (**d**) right-up, (**e**) right-down.

Figure 2 illustrates the process of shifted patch tokenization applied on the Type 2 colposcopy image dataset, while Figure 3 shows the shifted images of a sample Type 3 colposcopy image.

The primary difference between a regular Vision Transformer (ViT) and the one with patch tokenization is the way the input image is processed. In a regular ViT, the input image is typically split into non-overlapping patches, and each patch is treated as a sequence of flattened pixels, which are then fed into the transformer network [34]. The transformer network then processes these patches to learn relationships between different patches and make predictions.

**Figure 4:**
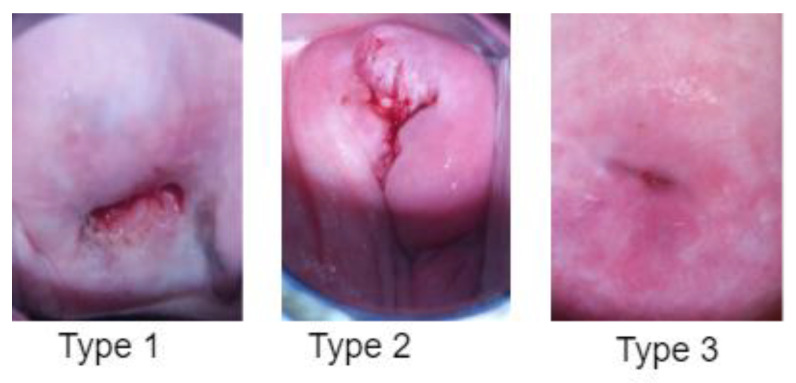
A sample of three different types of cervical pre-cancerous conditions from the dataset [15]. Type 1: Cervical intraepithelial neoplasia (CIN), Type 2: Squamous intraepithelial lesion (SIL), Type 3: Dysplasia.

In contrast, a ViT with patch tokenization further preprocesses, each patch using adding an additional “class” token at the beginning, similar to how a BERT [35] model processes text by adding a “start of sentence” token at the beginning of each sentence as shown in Figure 4. This class token represents the entire patch, and its embedding is learned along with the embeddings for the individual pixels within the patch.

By adding this class token, the ViT with patch tokenization is able to incorporate spatial information about the position of each patch within the image, which can improve its ability to recognize complex visual patterns. Additionally, using patch tokenization can reduce the number of patches required to represent an image, making the ViT more computationally efficient.

### 3.3. Dataset Description

The dataset used for training and testing the employed cervical cancer screening model consists of 8215 colposcopy images obtained from the public cervical screening data collection dataset by Intel and Smartphone ODT [15]. Different types of cervix were considered. The raw colposcopy images are classified by the experts, considering the transition zone visible in every image. These images include the three types of cervical pre-cancerous transformation zones. These images were then split into a 70:30 learning scheme, where 70% were used for training the network while the remaining images were used for evaluation purposes. A total of 5750 images were used for training and the rest for testing. Table 1 shows the learning scheme used for training and testing the models.

The images were all resized to 224 × 224 × 3 pixels for the reduction in computational costs. Figure 4 shows a sample of colposcopy images of the three different types of cervical pre-cancer conditions. In this study, we chose colposcopy as the primary modality for cervical cancer screening due to its distinct advantages over other available modalities. Colposcopy offers several unique features that align with the objectives of our research and provide a comprehensive assessment of cervical health. These reasons why we selected colposcopy over other modalities are as follows:

1. Direct Visual Examination: Colposcopy allows for a direct visual inspection of the cervix under magnification. This facilitates the identification of subtle morphological changes and abnormalities that might not be visible with other screening techniques.
2. Precise Localization: One of the key strengths of colposcopy is its ability to accurately localize abnormal areas on the cervix. This precise targeting is essential for guiding biopsies and subsequent interventions, ensuring accurate diagnosis and appropriate treatment.
3. Real-Time Assessment: Colposcopy provides a real-time evaluation of cervical tissue, enabling immediate decision-making regarding further investigations or interventions. This rapid assessment is critical for timely patient management.
4. Tissue Biopsy: Through colposcopy, targeted biopsies can be performed to obtain tissue samples from suspicious areas. This biopsy-guided approach enhances diagnostic accuracy and aids in determining the severity and nature of cervical abnormalities.
5. Clinician Expertise: Colposcopy is typically conducted by trained healthcare professionals with expertise in visual assessment. Their experience contributes to accurate interpretation and reduces the risk of misdiagnosis.

### 3.4. Evaluation Metrics

When evaluating a machine learning model, numerous metrics such as accuracy, precision, recall, and F1-score are used to analyze its performance. These metrics provide information about many aspects of the model’s generalization capabilities and help to determine its overall efficacy [16].

In this work, we used four different metrics to evaluate our model: Accuracy, Precision, Recall, and F1-Score.

Accuracy is a fundamental evaluation metric in classification tasks as it quantifies the proportion of correctly identified cervical types among all three types. However, accuracy alone may not always be enough to evaluate a model’s performance, especially when the classes are imbalanced, or the costs of false positives and false negatives fluctuate dramatically and can have a negative impact on the diagnosis results, especially in the medical field. Hence, we also used more metrics that opt to show the real performance of the employed model and for a fair comparison with the literature, in classifying the colposcopy images into three types.

(1) $$Accuracy=\frac{N}{T}$$

where *N* is the number of correctly identified colposcopy image types during testing, *T* is the total number of images used for testing the model.

Precision is a metric that measures the proportion of true positive predictions made by the model out of all positive predictions. It focuses on the accuracy of positive predictions, stressing the model’s capacity to avoid false positives. A high precision score indicates that the model is good at identifying positive cases while producing a few false alarms.

The fraction of true positive predictions out of all real positive instances in the dataset is measured by the recall, also known as sensitivity or true positive rate. Recall highlights the model’s capacity to correctly detect positive instances, ensuring that fewer positives are missed. A high recall score suggests that the model is good at capturing positive events and has a low rate of false negatives.

F1-score is a metric that combines precision and recall into a single metric to provide a balanced measure of the model’s performance. It is the harmonic mean of precision and recall and ranges from 0 to 1, with 1 being the highest attainable result. F1-score is especially beneficial when the dataset is skewed since it takes into account both false positives and false negatives.

Specificity is a measure that can indicate the accuracy of a test in correctly identifying those without a particular condition (true negatives).

The Mathew correlation coefficient (*MCC*) stands out as an optimal singular classification metric, serving to condense the information presented in a confusion matrix or an error matrix. Within a confusion matrix, four elements are encompassed.

(2) $$Precision=\frac{TP}{TP+FP}$$

(3) $$Recall=\frac{TP}{TP+FN}$$

(4) $$F1-score=2\times \frac{Precision\times Sensitivity}{Precision+Sensitivity}$$

(5) $$Specificity=\frac{TN}{TN+FP\;}$$

(6) $$MCC=\frac{TN\times TP-FN\times FP}{\sqrt{\left(TP+FP\right)\left(TP+FN\right)\left(TN+FP\right)\left(TN+FN\right)}}$$

where *TP* (True Positive) represents the count of correctly predicted positive instances, while *TN* (True Negative) represents the count of correctly predicted negative instances. On the other hand, *FP* (False Positive) indicates the count of wrongly predicted positive instances, and *FN* (False Negative) indicates the count of wrongly predicted negative instances.

## 4. Model Development and Parameters

Several critical phases were involved in developing our vision Transformer (ViT) with shifting patch tokenization for classifying cervical pre-cancerous colposcopy images into three types. First, images were collected, reprocessed, and divided into their appropriate types. To maintain uniformity in the input data, the images were scaled to a constant resolution of 224 × 224 × 3 pixels. The ViT architecture is then built, following the basic 16 × 16 patches size structure with one Encoder block. This structure separates the input image into equal-sized patches, each of which represents a token in addition to the class token which is added at the beginning of the input sequence and carries information about the whole image. During the self-attention computation in the transformer layers, the class token interacts with the patch tokens, allowing the model to attend to relevant features and make predictions based on the global context of the image. The patch tokens are then supplied into the Transformer model, along with their positional encodings. However, in our case of shifted patch tokenization, a modification is introduced to enhance the Transformer’s ability to capture spatial information. In contrast to the regular ViT which uses non-overlapping patches, the patches in our case are shifted by a certain stride to partially overlap. This allows the model to capture contextual information across neighboring patches, thus improving its understanding of spatial relationships in the image.

**Figure 5:**
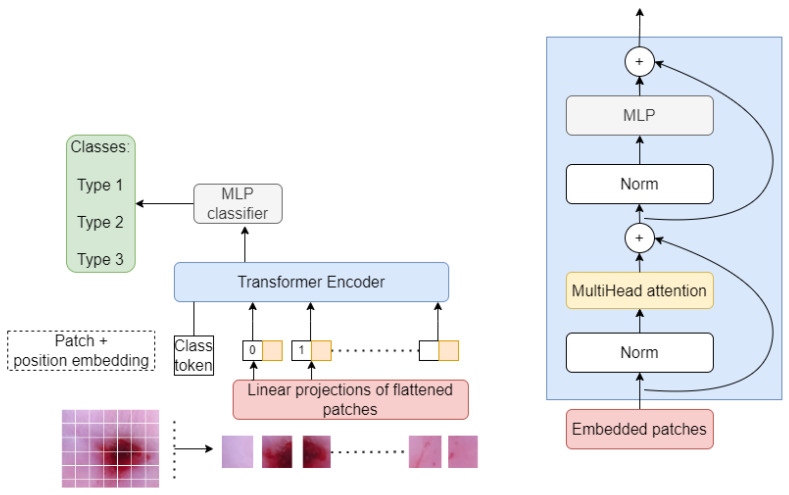
The vision Transformer architecture for classifying cervical colposcopy images into three types.

Figure 5 shows the Vision Transformer with shifted patched tokenization architecture. As seen, our Transformer encoder consists of a multi-head attention mechanism, normalization layers, and multilayer perceptron (MLP). The output of this encoder is then passed through a feed-forward neural network, which allows the model to learn correlations and patterns in the image and classify them using SoftMax activation function.

## 5. Results

The training procedure begins once the model architecture is defined. Using a stratified sample strategy, the labeled dataset is divided into training and validation sets. The training set is used to iteratively optimize the model’s parameters. The goal of optimization is to minimize a preset loss function, categorical cross-entropy, which quantifies the difference between the predicted and true labels. It was critical to divide the dataset into training and testing phases. All divisions were carried out with the goal of dividing the three different colposcopy classes as evenly as feasible. As a result, data leakage and imbalance between the training and testing sets are avoided. The training step was repeated for each hyperparameter combination that was generated during the subsequent optimization phase. The purpose of hyperparameter adjustment was to increase model efficacy and decrease classification errors. The dataset was divided into 70% training and 30% testing. It should be noted that the training and testing pipelines for the developed ViT model were built using the TensorFlow 2.5 framework.

The Adam optimization method has been shown to outperform its competitors among those now available. As a consequence, the Adam optimization strategy with a gradient decay value of 0.9 was utilized to train the model. The initial learning rate was set to 0.001 and the regularization factor was set at 0.0001. The model was eventually trained for 100 epochs with a minibatch size of 64 due to memory restrictions.

**Figure 6:**
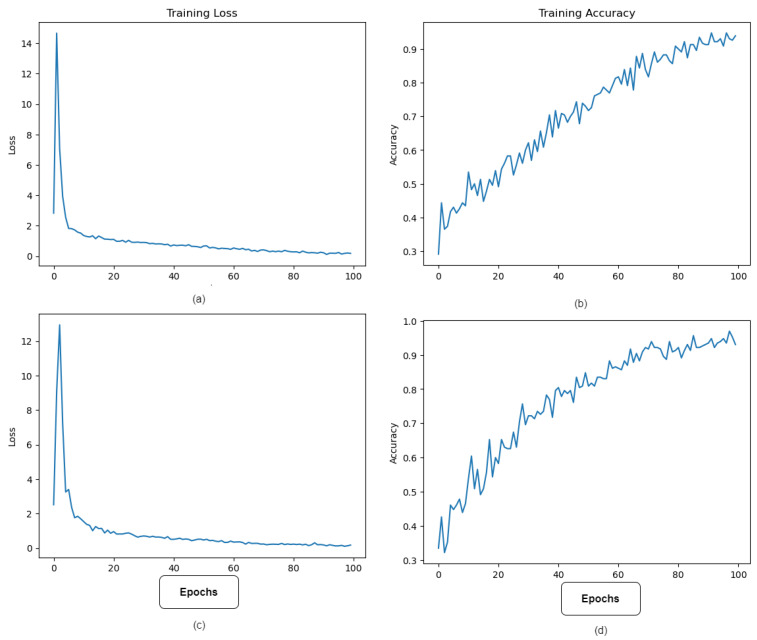
Accuracy and loss variations during the training process. (**a**,**b**) show the loss and accuracy variations over the number of epochs of the ViT model, while (**c**,**d**) show the loss and accuracy change in the improved ViT with SPT.

Figure 6 depicts the best model performance’s training accuracy and loss. The model’s lowest error occurred at epoch 100, when learning halted due to the implementation of the Early Stopping method during training to prevent overfitting.

**Figure 7:**
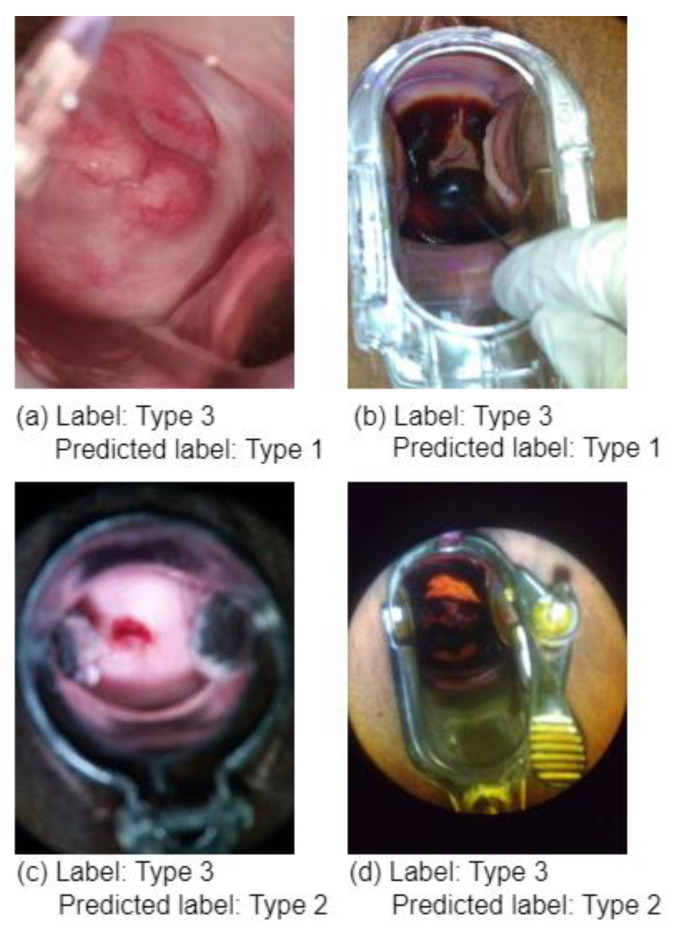
A sample of incorrectly predicted type 3 colposcopy images: (**a**) Type 3 image predicted as Type 1, (**b**) Type 3 images predicted as Type 1, (**c**) Type 3 images predicted as Type 2, (**d**) Type 3 images predicted as Type 2.

As previously stated, the model was evaluated on 30% of the data, and to demonstrate the practicality of the SPT, we also trained and tested a regular ViT without SPT on the same dataset. The testing results of the regular ViT and the ViT augmented with shifting patch tokenization and location self-attention are shown in Table 2. It is widely acknowledged that the use of SPT increased the ViT’s performance. Figure 7 shows some type 3 colposcopy images which were incorrectly predicted as Type 1 and Type 2. The reason the Type 3 class has more incorrectly classified images than other classes could be that this class has more complex images where devices or other objects (metal objects) are included in the image which makes it hard for the model to extract the relevant features to such class. Moreover, this can be due to the complexity and similarity of Type 3 compared to Type 1 and 2 colposcopy images. Figure 7 shows some of the incorrectly predicted colposcopy images.

**Figure 8:**
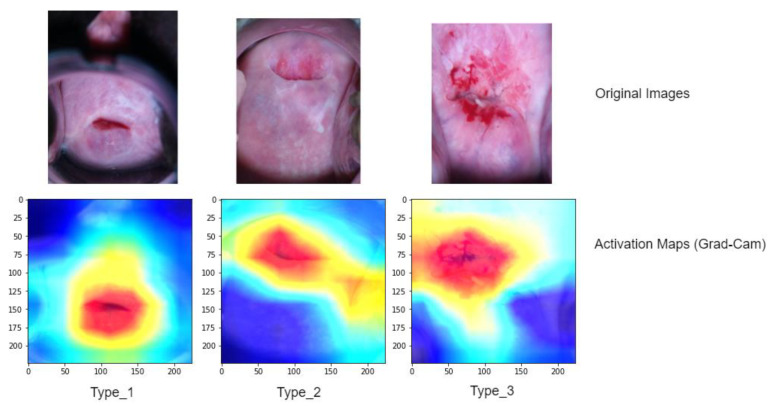
The Grad-CAM technique, based on the ViT with SPT (Shifted Patch Tokenization), was utilized to obtain localizations on testing cervical colposcopy images. In the presented visualizations, the first row showcases the original images, while the second row displays the corresponding classification activation maps overlaid on the images. These activation maps highlight the regions within the images that contributed most significantly to the classification decision made by the model. The colors in a Grad-CAM heatmap usually range from red-to-blue color scale, where red signifies the highest importance and blue signifies the lowest importance. The intensity of the color represents the degree of importance.

To gain a better understanding of the model’s performance, we further analyze activation maps that indicate the specific areas the model concentrated on while making grading decisions for each image (refer to Figure 8). To compute and visualize these activations, we utilized a technique called gradient weight class activation mapping (Grad-Cam). These activation maps employ heatmaps, where regions suspected to be associated with a predicted class are displayed using a jet colormap. In this colormap, the areas with the highest activation are depicted as deep red, while the areas with the lowest activation are shown as deep blue. 

## 6. Discussion

### 6.1. Results of Comparison

Cervical cancer is the major cause of cancer death in poor nations among women [1]. The condition can be effectively treated if detected early [3]. As a result, computerized cervical screening to diagnose the transformation zone has a significant clinical impact in underdeveloped nations, particularly in locations where medical resources are sparse.

Cervical cancer screening is considered a critical task as it benefits patients to predefine their pre-cancerous transformation zone and find the suitable treatment at the right time [1,5]. A growing number of researchers have produced promising results when using deep learning technologies to classify cervical cancer [7,36,37,38] or to help in predicting its occurrence [37,38]. However, there are large discrepancies in classification performance among researchers, with accuracy, sensitivity, and specificity ranging from 50 to 99, 60–98, and 70–98, respectively [7,37,38].

This study aims to introduce a simple, yet effective Vision Transformer-based model trained to surpass the performance of other complex architectures proposed for identifying cervical pre-cancerous types from colposcopy raw images. Our approach involved enhancing the ViT architecture with SPT, which resulted in an impressive overall accuracy of 91.02%, precision of 91%, and F1-score of 94% (as shown in Table 2) for classifying cervical pre-cancerous types. This performance is highly promising, as our model achieved comparable results to other related studies (as demonstrated in Table 3) when considering accuracy as the comparative metric, which was the most commonly reported metric in previous studies.

Furthermore, the results presented in Table 3 demonstrate that our trained Transformer model’s accuracy aligns with state-of-the-art studies in grading cervical pre-cancerous colposcopy images. Despite the simplicity of our proposed transfer learning approach, we have demonstrated that with a well-curated dataset and a sophisticated shifted patch tokenization technique applied to input images, Vision Transformer can be successfully utilized in medical image diagnostic research. In the pursuit of innovative solutions, this study presents a pioneering approach to cervical cancer pre-screening. The core novelty resides in the strategic fusion of two powerful technologies: improved vision transformers and shifted patch tokenization. The synergy of these elements culminates in an unprecedented methodology for classifying three distinct types of cervical pre-cancerous colposcopy images.

The primary contribution of this study lies in the creative adaptation of vision transformers—a technology initially formulated for image classification tasks—into the realm of cervical cancer diagnosis. By enhancing these transformers with a shifted patch tokenization mechanism, a finer granularity of analysis is achieved. This granular insight allows for the classification of subtle differences among pre-cancerous colposcopy images, a feat that was previously challenging with traditional methodologies.

In summary, our work offers the following results:

- Surpassing the performance of several state-of-the-art techniques used for automatic grading of cervical pre-cancer types from colposcopy images, achieving higher accuracy compared to previous studies [7,37,38] conducted on two different colposcopy datasets.
- Demonstrating that selecting an appropriate, efficient, yet simple model architecture can yield better results than relying on highly complex architectures [23,37,38], or using transfer learning for grading cervical pre-cancer types.
- Presenting the activation map using Grad-Cam, which serves as an additional tool for diagnosing KOA (knee osteoarthritis).
- Publicly releasing our model architecture to ensure reproducibility and facilitate further research in the field.

### 6.2. Limitations and Challenges

The primary goal of this CAD is to assist colposcopists in improving their diagnostic abilities, not to replace them. The CAD diagnosis result is viewed as a “second set of eyes” of human colposcopists, and human colposcopists are ultimately responsible for the final diagnosis result. Despite the computer-aided diagnosis (CAD) excellent performance in colposcopy imaging, there are still significant problems and obstacles to overcome. Colposcopy raw images for patients with cervical canal lesions and Type 3 transformation zone must still be taken in more appropriate methods where no gadgets or other objects can be visible.

In this work, we analyzed three types of transformation zone which all have some very similar features, particularly Type 3 which was challenging for the model to be graded distinctly due to the complexity of the images found in that category and the similarity between its images and images from other types. Despite the multi-head attention of our model, it is still a data hungry model and to grasp the features needed for making the optimum possible efficiency, such model still needs a huge number of images to train. We attempted to solve this problem by embedding the shifted patch tokenization techniques into our model architecture which helped in achieving better accuracy with a relatively small dataset, however training this model on a larger dataset can more likely lead to a better generalization capability which can tackle some challenges faced during testing such as identifying images with metal objects inside, or similar features images (Type 3).

**Figure 9:**
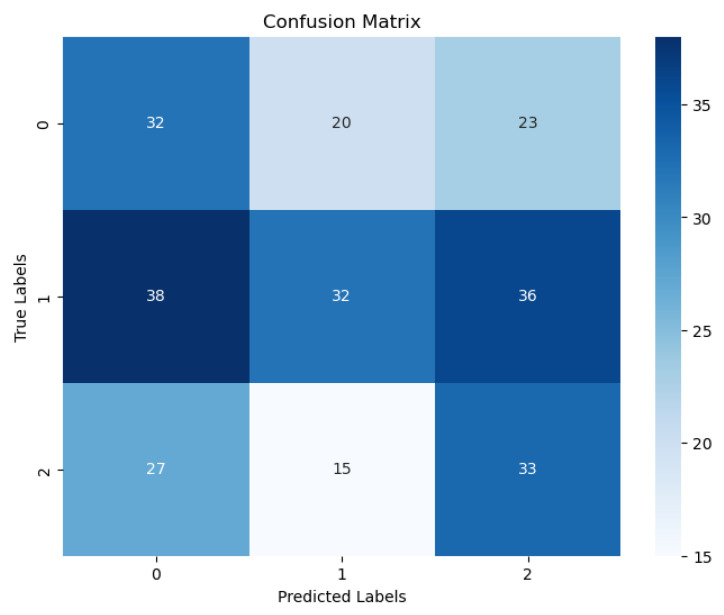
Confusion matrix of the best result for our three classes ViT with SPT model.

**Figure 10:**
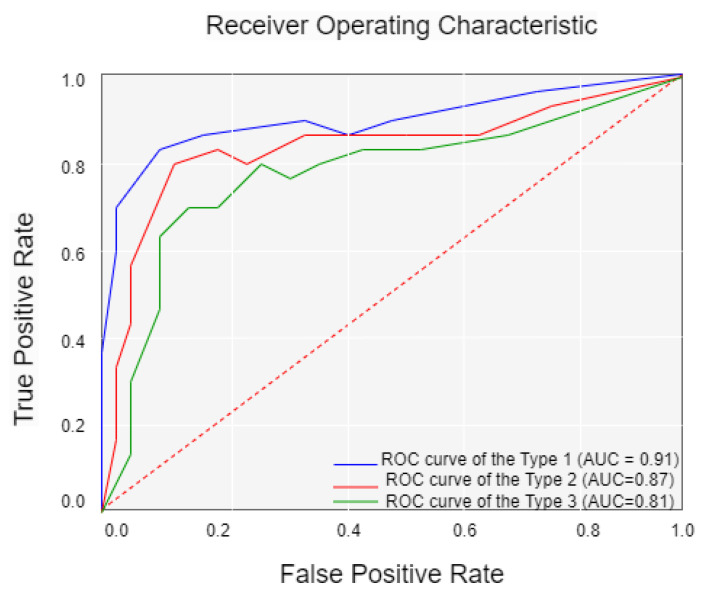
Receiver operating character (ROC) and area under curve (AUC) of the three cervical colposcopy types.

Finally, this study is retrospective research. Prospective studies are required to validate the performance of our model. Fourth, the clinical characteristics we included in the study were insufficient; smoking history, age at first sex, and number of sexual partners should also be considered. Figure 9 shows the confusion matrix of the best result achieved by our ViT with the SPT model. Figure 10 shows the Receiver Operating Character (ROC) and Area Under Curve (AUC) of the three cervical colposcopy Types.

## 7. Conclusions

In this study, we created a Vision Transformer-based classification system that can assist colposcopists in recognizing cervical precancerous colposcopy image types. Despite the small dataset on which the model was trained, our model architecture incorporated the use of shifting patch tokenization, which helped improve its performance. The final evaluation results of the model showcased the potential for providing an objective diagnostic foundation for colposcopists and yielding clinical application value. Moving forward, our future endeavors will involve collecting multicenter data and conducting more comprehensive research. The aim is to further refine and adapt this model for clinical practice by incorporating additional metrics alongside the colposcopy images. This expansion will enable a more comprehensive and robust analysis, enhancing the model’s overall utility and effectiveness in real-world medical settings.

## Figures and Tables

**Table 1:** Total number of images collected from the dataset.

	Ratio	Total Number of Images
Total	100%	8215
Train	70%	5750
Test	30%	2464

**Table 2:** The performance of the model during testing.

	ViT with SPT and LSA
Accuracy	91.02%
Precision	91%
Specificity	90%
Recall	92%
F1-score	94%
Mathew Coefficient	0.82

**Table 3:** Comparative results.

	Method	Dataset	Accuracy
Mustafa and Dauda (2019) [23]	Adam based CNN	Raw colposcopy images	90%
Yuan et al. (2020) [7]	ResNet	Histopathological	84.1%
Liu et al. (2021) [37]	ResNet	Raw colposcopy images	80.7%
Peng et al. (2020) [38]	ResNet	Raw colposcopy images	80.4%
Peng et al. (2020) [38]	DenseNet	Raw colposcopy images	76.4%
Peng et al. (2020) [38]	VGG16	Raw colposcopy images	86.3%
Current research (Our paper)	ViT with SPT	Raw colposcopy images	91%

## Data Availability

The dataset explored in the research can be found at https://www.kaggle.com/c/intel-mobileodt-cervical-cancer-screening/data, accessed on 4 September 2023.
